# Validation of an ecological momentary assessment to measure processing speed and executive function in schizophrenia

**DOI:** 10.1038/s41537-021-00194-9

**Published:** 2021-12-21

**Authors:** Cecelia Shvetz, Feng Gu, Jessica Drodge, John Torous, Synthia Guimond

**Affiliations:** 1 The Royal’s Institute of Mental Health Research, Ottawa, ON Canada; 2 Department of Cellular and Molecular Medicine, University of Ottawa, Ottawa, ON Canada; 3 Department of Psychiatry and Division of Clinical Informatics, Beth Israel Deaconess Medical Center, Harvard Medical School, Boston, MA USA; 4 Department of Psychiatry, University of Ottawa, Ottawa, ON Canada; 5 Département de Psychoéducation et Psychologie, Université du Québec en Outaouais, Gatineau, QC Canada

**Keywords:** Human behaviour, Schizophrenia

## Abstract

Cognitive impairments are a core feature of schizophrenia that have negative impacts on functional outcomes. However, it remains challenging to assess these impairments in clinical settings. Smartphone apps provide the opportunity to measure cognitive impairments in an accessible way; however, more research is needed to validate these cognitive assessments in schizophrenia. We assessed the initial accessibility, validity, and reliability of a smartphone-based cognitive test to measure cognition in schizophrenia. A total of 29 individuals with schizophrenia and 34 controls were included in the analyses. Participants completed the standard pen-and-paper Trail Making Tests (TMT) A and B, and smartphone-based versions, Jewels Trail Tests (JTT) A and B, at the single in-lab visit. Participants were asked to complete the JTT remotely once per week for three months. We also investigated how subjective sleep quality and mood may affect cognitive performance longitudinally. In-lab and remote JTT scores moderately and positively correlated with in-lab TMT scores. Moderate test-retest reliability was observed across the in-lab, first remote, and last remote completion times of the JTT. Additionally, individuals with schizophrenia had significantly lower performance compared to controls on both the in-lab JTT and TMT. Self-reported mood had a significant effect on JTT A performance over time but no other significant relationships were found remotely. Our results support the initial accessibility, validity and reliability of using the JTT to measure cognition in schizophrenia. Future research to develop additional smartphone-based cognitive tests as well as with larger samples and in other psychiatric populations are warranted.

## Introduction

Schizophrenia is a chronically disabling psychiatric disorder estimated to affect 20 million people worldwide^1^. Cognitive impairments are a core feature of schizophrenia and are known to impact social and occupational functioning^2,3^. While cognitive impairments influence the quality of life of individuals with schizophrenia, several factors limit their assessment in clinical settings^4^.

Current assessments of cognition are limited to time and resources as they require highly trained professionals for administration, and take a long time to complete^4,5^. Consequently, cognitive assessments in psychiatry tend to rely heavily on retrospective self-reports which can be biaised^6^. Moreover, compliance to therapy and scheduled appointments is generally low in schizophrenia^7^. Hence, long appointment duration for neuropsychological testing is not ideal for this population. Cognitive assessments that incorporate technology-based interventions could therefore be helpful to improve patient engagement^8^.

Computerized assessments of cognition such as the Cogstate (https:// cogstate.com) and the Cambridge Neuropsychological Test Automated Battery (CANTAB; http: www.cambridgecognition.com/cantab/) have been used for some time with people with schizophrenia^9–11^. Computer-based assessments are capable of offering more standardized testing with automated scoring which is particularly advantageous when multiple testing sessions are required^12–14^. Smartphone-based cognitive tests introduce an array of benefits over their computer-based counterparts. Smartphone assessments are not only portable and available at an individual’s fingertips, but they also introduce the ability to collect dynamic data as it relates to cognition such as exercise, sleep, mood, and more^15^. Smartphone assessments can be completed virtually anywhere at any time^16^, and therefore allow for brief assessments of cognition, otherwise known as ecological momentary assessments (EMAs)^17^.

Increasing smartphone ownership and usage is observed across all ages and generations in both developed and developing societies^18,19^. Smartphone assessments are also well received by individuals with schizophrenia who are interested in using digital data to augment their care^20^. A recent randomized control study reported that 82.1% of their participants with schizophrenia owned a smartphone^21^. Therefore, smartphone apps may prove to be an accessible and valid mean of assessing cognition in this population^15^.

Of the cognitive testing apps currently available, most are profit-oriented, requiring monthly subscriptions or in-app purchases, and remain to be validated through research (e.g., Savonix Mobile, https://savonix.com; CogniSense, http://www.questcognisense.com). A recent study provided pioneering evidence on the validity of a cognitive EMA using a smartphone app targeting a specific cognitive domain (i.e., verbal memory) for people with a serious mental illness, such as schizophrenia^22^. In this study, smartphones were provided to participants. Although clinical trials are often capable of providing smartphone devices to their participants, this would not be the case in non-research settings, such as mental health clinics. This study also did not include any analysis of test-retest reliability which is important in the interpretation of a validation study of a new cognitive test. Hence, further investigation of open-access EMAs of additional cognitive domains impaired in schizophrenia and reporting on ecological accessibility and test-retest reliability are needed.

**Fig. 1:**
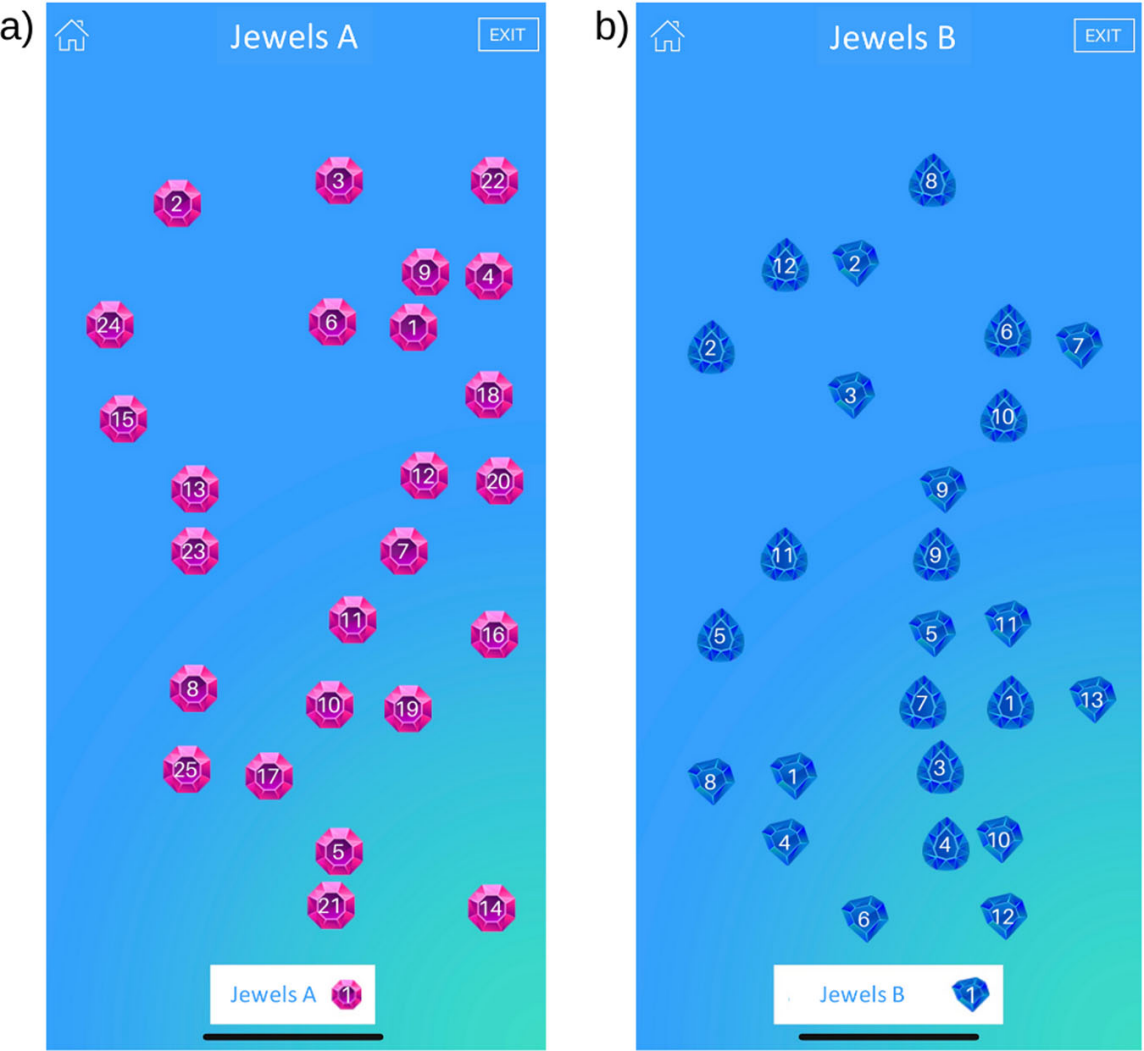
JTT smartphone-based cognitive assessments on the mindLAMP app. **a** On the JTT A, participants tap on the numbers in chronological order, **b** on the JTT B; participants alternate between numbers and letters in chronological order.

Our team has recently developed a smartphone app named mindLAMP that is open source and free for anyone to use^23^. This app was built with a patient-centered approach, intending to monitor, manage and prevent chronic mental illnesses. The mindLAMP app also enabled the development of two cognitive assessments called the Jewels Trail Tests (JTT) A and B (see Fig. 1) which are modelled off the gold-standard Trail Making Tests (TMT) A and B^24,25^. The TMT A and B measure processing speed and executive functioning, respectively; two cognitive domains that are frequently impaired in schizophrenia^3,26^. The TMT are commonly used in cognitive batteries for schizophrenia which made them great model candidates for the smartphone JTT development^27,28^. The main difference between the JTT and TMT is that in order to make the tests usable and engaging on a smartphone, users tap the numbers instead of drawing a line between them. They also have to alternate between two different shapes instead of numbers and letters for the part B. Preliminary evidence shows that it is feasible to use these mindLAMP cognitive tests and that they may differentiate people with schizophrenia from controls^29^. However, the tests remain to be validated to assess actual cognitive impairments in schizophrenia.

We believe that the JTT offered through the mindLAMP app could serve a multitude of purposes. Firstly, they could be used in research settings to measure cognitive performance in-lab and remotely over time. Through the mindLAMP app, the JTT can be paired with surveys and questionnaires, making it easy to explore factors that influence cognition over time such as symptomatology, sleep, and exercise. The JTT could also be used as a substitute or addition to current clinical assessments of cognition which could increase rates of cognitive testing in clinical settings without increasing the demand for resources such as time and trained staff. They could also be used to measure cognitive side effects around psychotropic medications, especially when starting and tapering medications. Moreover, the JTT could serve as an effective way to continue clinical, and research based cognitive assessments during the ongoing COVID-19 pandemic by reducing face-to-face contact as the tests can be administered virtually anywhere. In the future, the mindLAMP app and JTT could also be used as an educational tool and provide key information to patients and their support teams, such as tracking cognitive performance over time. Hence, there is a need to validate the JTT and explore the fluctuations in cognitive performance over time in various clinical populations with cognitive impairments, such as schizophrenia.

In the present study, we assessed the initial accessibility, validity, and reliability of the mindLAMP app to measure processing speed and executive functioning in schizophrenia. We hypothesized that the app would be accessible and well accepted by participants. We also hypothesized that cognitive performance scores on the JTT would show convergent validity and positively correlate with the scores obtained on the standardized, pen-and-paper TMT. We hypothesized that results would show moderate test-retest reliability of the JTT. We also investigated between-group differences and hypothesized that the JTT would show similar sensitivity and specificity as the TMT at discriminating between individuals with schizophrenia and controls. Finally, we explored how self-reported mood and subjective sleep quality may influence cognitive performance on the JTT on a weekly basis and hypothesized that cognitive performance on both the JTT A and B would be affected.

## Results

### Demographic data

Demographic and clinical data from the final sample are presented in Table 1. The specific demographic and clinical data from the sample included in the in-lab baseline analyses are presented in Supplementary Table 1. We observed no statistically significant differences between our groups in terms of age, sex, and smartphone use.

**Table 1:** Demographic and clinical data for schizophrenia and control groups from the final sample.

	Schizophrenia group (*N* = 29)	Control group (*N* = 34)	*p*-value
Age (years)			0.08
Mean (SD)	38.0 (11.3)	32.3 (11.3)	
Median [Min, Max]	37.0 [20.0, 64.0]	29.0 [18.0, 59.0]	
Sex			0.07
Male	24.0 (82.8 %)	20.0 (58.8 %)	
Female	5.0 (17.2 %)	14.0 (41.2 %)	
Smartphone use			0.97
Every day of the week	28.0 (96.6%)	33.0 (97.1 %)	
6 or less days per week	1.0 (3.4%)	1.0 (2.9 %)	
PANSS Positive Score			
Mean (SD)	15.9 (5.7)		
Median [Min, Max]	16.0 [7.0, 28.0]		
PANSS Negative Score			
Mean (SD)	17.5 (6.9)		
Median [Min, Max]	16.0 [8.0, 33.0]		
PANSS General Score			
Mean (SD)	31.6 (7.8)		
Median [Min, Max]	31.0 [20.0, 52.0]		
CPZ-equivalence			
Mean (SD)	501.8 (402.4)		
Median [Min, Max]	400.0 [75.0, 2100.0]		

CPZ-equivalence was calculated for all antipsychotics based on the equivalent oral dose of chlorpromazine.

*SD* standard deviation, *PANSS* Positive and Negative Syndrome Scale, *CPZ equivalence* chlorpromazine-equivalence.

### Accessibility results

A total of 86.56% of individuals with initial interest in participating in the study were able and interested to download the mindLAMP app to their phones. More specifically, out of the 119 people who contacted us with initial interest in participating in the study, only 8.40% (9 SZ and 1 HC) could not participate because they did not own a smartphone. An additional 5.04% (1 SZ and 5 HC) were not interested in downloading the app to their phone and therefore declined to participate. The other interested individuals who were not recruited for the study were found to not meet inclusion/exclusion criteria following the phone screening interview.

Over the three-month study period, participants completed the JTT A an average of 5.05 times (SD = 4.34, range = 1–17) and the JTT B an average of 4.97 times (SD = 4.27, range = 1–17). A total of 8 individuals (4 SZ and 4 HC) completed the JTT A 12 times or more and 7 (3 SZ and 4 HC) completed the JTT B 12 times or more, whereas 18 participants (7 SZ and 11 HC) never completed the JTT A remotely and 21 (9 SZ and 12 HC) never completed the JTT B remotely.

### Validity

**Fig. 2:**
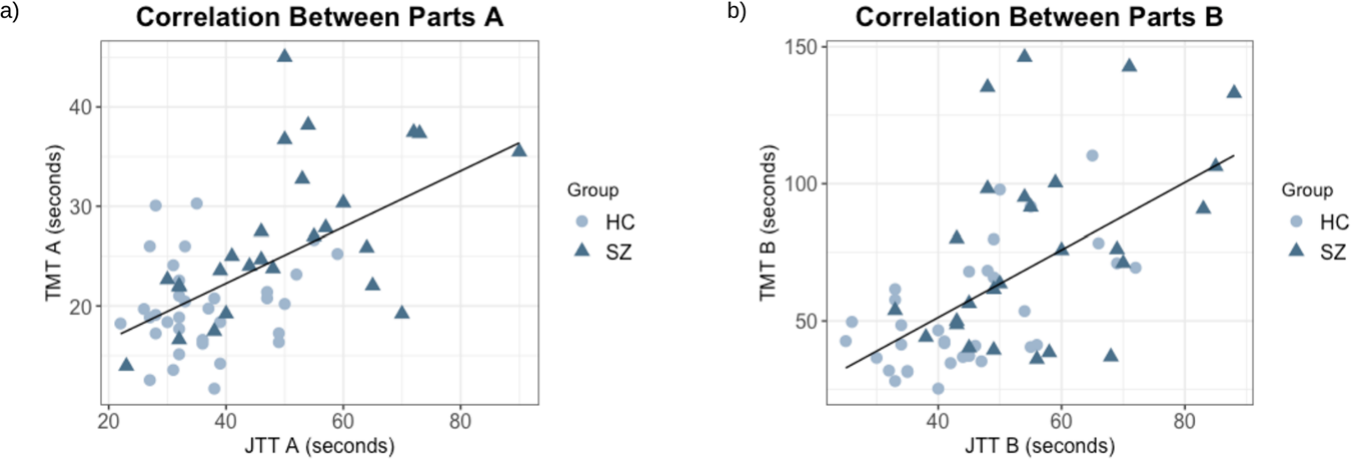
Relationship between performance on smartphone app and pen-and-paper in-lab cognitive assessments at baseline. **a** JTT A is positively correlated with TMT A; **b** JTT B is positively correlated with TMT B. HC Healthy controls, SZ schizophrenia, JTT Jewels Trail Tests, TMT Trail Making Tests. Cognitive performance was measured in seconds where a greater completion time represents a worse score.

Results indicate significant, moderate, and positive relationships between in-lab performance on the standardized pen-and-paper TMT and the smartphone app JTT on both Parts A (*r* = 0.57, *p* < 0.001, 95% CI [0.37, 0.72]*)* and B (*r* = 0.58, *p* < 0.001, 95% CI [0.38, 0.73]*)* (See Fig. 2). These results were not significantly different between groups (all *p* ≥ 0.103). Participants were also completing the TMT A in-lab (*M* = 22.93, SD = 6.99) significantly faster than the JTT A in-lab (*M* = 42.35, SD = 14.21) (*t*_(86)_ = −9.50, *p* < .001, 95% CI [−23.48, −15.36]) and the TMT B (*M* = 63.12, SD = 30.25) significantly slower than the JTT B (*M* = 49.63, SD = 14.26) (*t*_(84)_ = 3.12, *p* = 0.002, 95% CI [4.90, 22.07]).

We also observed significant, moderate, and positive correlations between the last remote JTT and the in-lab JTT (A: *r* = 0.56*, p* < 0.001, 95% CI [0.29, 0.75]; B: *r* = 0.41*, p* = 0.012, 95% CI [0.10, 0.65]) as well as between the last remote JTT and the in-lab TMT (A: *r* = 0.66*, p* < 0.001, 95% CI [0.42, 0.81]; B: *r* = 0.33, *p* = 0.046, 95% CI [0.01, 0.60]). These results were not significantly different between groups (all *p* ≥ 0.294).

### Test-retest reliability

We observed moderate reliability over time within participants on both the JTT A (*ICC* = .60, *p* < 0.001, 95% CI [0.45, 0.73]) and JTT B (*ICC* = .55, *p* < 0.001, 95% CI [0.39, 0.69]). Furthermore, a low percentage of overall group variance was observed between the in-lab and first-remote assessments for both the JTT A (3.40%) and B (12.20%).

### Between-group differences

**Fig. 3:**
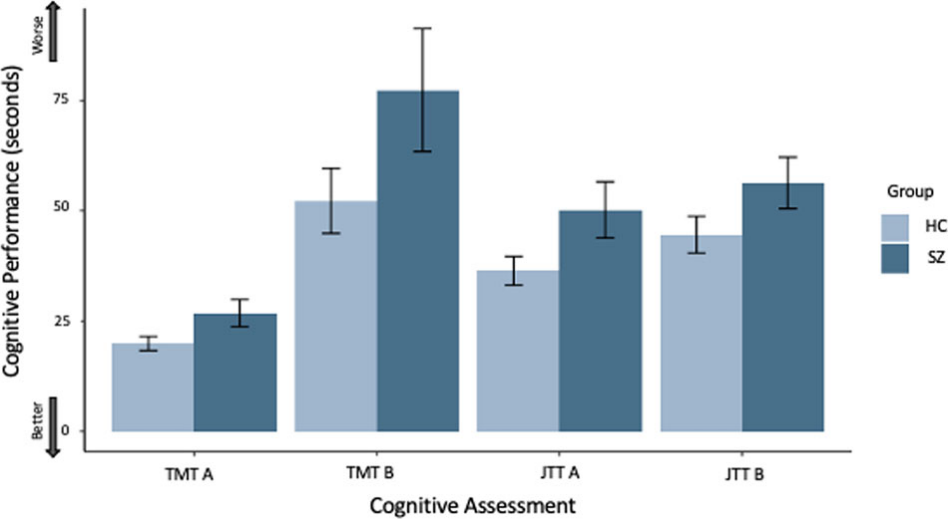
Differences in cognitive performance between individuals with schizophrenia and healthy controls on in-lab cognitive assessments. JTT Jewels Trail Tests, TMT Trail Making Tests, HC healthy controls, SZ individuals with schizophrenia/schizoaffective disorder. All comparisons survive Bonferroni corrections *p* < 0.05. Error bars represent 95% confidence intervals. Cognitive performance was measured in seconds where a greater completion time represents a poorer score.

Table 2 and Fig. 3 show the between-group results for the cognitive performance across all four in-lab tests at baseline. We observed significant between-group differences on all tests (JTT A: *B* = 11.51, *SE* = 3.11, *β* = 0.40, *t*_(56)_ = 3.71, *p* < 0.001, 95% CI [5.29, 17.73]; JTT B: *B* = 9.98, *SE* = 3.48, *β* = 0.35, *t*_(56)_ = 2.87, *p* = 0.006, 95% CI [3.00, 16.95]; TMT A: *B* = 6.39, *SE* = 1.68, *β* = 0.46, *t*_(56)_ = 3.81, *p* < 0.001, 95% CI [3.03, 9.75]; TMT B: *B* = 24.41, *SE* = 7.53, *β* = 0.40, *t*_(56)_ = 3.24, *p* = 0.002, 95% CI [9.31, 39.50]). Individuals with schizophrenia had significantly poorer cognitive performance on all in-lab tests compared to controls.

**Table 2:** Means and standard deviations for cognitive performance in schizophrenia and control groups on in-lab JTT and TMT at baseline.

	JTT A	JTT B	TMT A	TMT B
SZ				
Mean (*SD*)	50.15 (15.81)	56.31 (14.46)	26.83 (7.76)	77.37 (34.52)
HC				
Mean (*SD*)	36.38 (9.31)	44.53 (11.98)	19.95 (4.55)	52.22 (21.23)

Cognitive performance represents time in seconds to complete the test. Cognitive performance was measured in seconds where a greater completion time represents a poorer score.

*TMT* Trail Making Test, *SD* standard deviation, *SZ* schizophrenia, *HC* healthy controls.

We also observed a main effect of age on the JTT A (*B* = 0.49, *SE* = 0.13, *β* = 0.40, *t*_(56)_ = 3.65, *p* < 0.001, 95% CI [0.22, 0.76]) as well as a trending effect of age on the JTT B (*B* = 0.30, *SE* = 0.15, *β* = 0.24, *t*_(56)_ = 1.98, *p* = 0.053, 95% CI [−0.00, 0.60]). Specifically, as age increased, the cognitive performance scores on the JTT also increased, indicating poorer cognition. No significant effects of age were found on the TMT A or B (all *p* ≥ 0.190). No significant sex differences were observed on any of the in-lab cognitive assessments (all *p* ≥ 0.431).

**Fig. 4:**
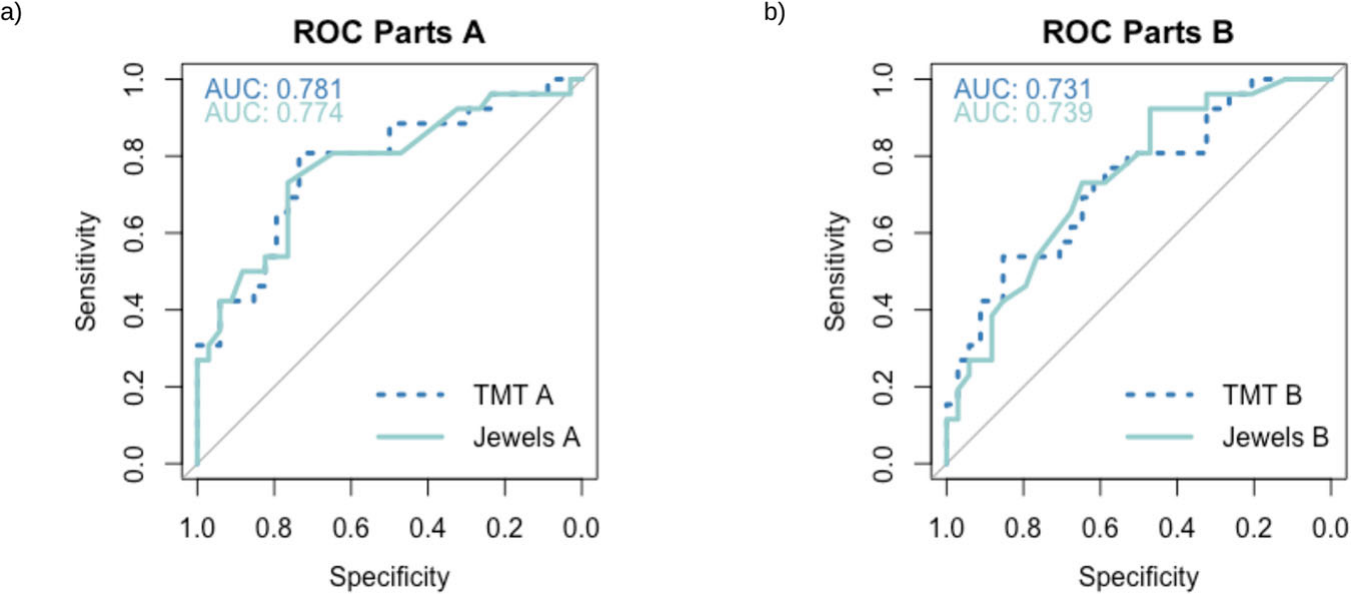
Receiver operating curves results comparing the sensitivity and specificity for both the smartphone app JTT and pen-and-paper TMT. **a** JTT A and TMT A show similar sensitivity and specificity; **b** JTT B and TMT B show similar sensitivity and specificity. ROC receiver operating characteristic curves, AUC area under the curve, TMT Trail Making Test, JTT Jewels Trail Tests.

Receiver operating curves show the sensitivity and specificity of the JTT in comparison to the TMT from the in-lab assessment. Specifically, the area under the curves from the receiver operating characteristic analysis demonstrated that the JTT were as sensitive and as specific as the standardized TMT at discriminating between groups (see Fig. 4).

### Longitudinal analyses

No significant practice effects were observed on either JTT (A: (*B* = −234.97, *SE* = 162.82, *β* = −0.07, *t*_(288.30)_ = −1.44, *p* = 0.150, 95% CI [−0.16, 0.02; B: (*B* = −320.73, *SE* = 186.35, *β* = −0.08, *t*_(273.02)_ = −1.72, *p* = 0.086, 95% CI [−0.17, 0.01]). We observed that self-reported mood significantly affected cognitive performance on the JTT A over time (*B* = −881.80, *SE* = 349.90, *β* = −0.14, *t*_(283.90)_ = −2.52, *p* = 0.012, 95% CI [−0.25, −0.03]). This relationship was negative where better self-reported mood was related to faster completion times on the JTT A (i.e., better cognitive performance). Significant effect was found neither between self-reported mood and the JTT B (*p* = 0.243), nor between subjective sleep quality and either the JTT A or B (all *p* ≥ 0.277). Lastly, no significant between-group interactions were found with any of the associations between mood nor subjective sleep quality and the JTT A or B (all *p* ≥ 0.291).

### Exploratory analyses

No significant relationship was observed between screen size and cognitive performance on the JTT in-lab (all *p* ≥ 0.580) Significant relationships were initially observed between the PANSS positive subscore and the JTT A in-lab (*r* = 0.40, *p* = 0.044, 95% CI [0.01, 0.68]) and the PANSS negative subscore and the JTT B in-lab (*r* = 0.42, *p* = 0.034, 95% CI [0.04, 0.69]), but these results did not survive Bonferroni corrections (all *p* ≥ 0.05 / 6 tests = 0.008). No other significant relationships were found between JTT B in-lab and the positive subscore, the JTT A in-lab and the negative subscore, nor general subscores (all *p* ≥ 0.204). Finally, no significant associations were observed between the number of completion times of the JTT over time and any demographic characteristics, including sex, age, education, and diagnostic group (all *p* ≥ 0.348).

## Discussion

This study aimed to validate an EMA assessing processing speed and executive functions in schizophrenia^22^. Our findings indicate that mindLAMP is a promising and well accepted EMA tool to assess cognitive performance in individuals with schizophrenia. We chose to only recruit individuals who already owned a smartphone as we wanted to test the mindLAMP app on varying smartphone makes, models, and operating systems, and to assess the potential of the app being used in settings where smartphones are not provided to the individual such as mental health clinics. Our results indicated that the app was both accessible and accepted by SZ and HC participants. We found that the majority of our recruited participants were interested and able to use the app on their smartphones. We also observed significant, positive and moderate correlations between cognitive performances on the app and the standardized pen-and-paper TMT which support the convergent validity of the JTT in-lab and remotely over time.

Our findings highlight that the JTT work in a similar way to the gold-standard TMT, which is widely used to measure processing speed and executive functioning in schizophrenia^28,30^. The results also suggest that the JTT could be used in a similar manner as the TMT to identify these cognitive impairments in the schizophrenia population. We observed significant between-group differences showing that schizophrenia participants consistently had lower cognitive performance across all test forms. The JTT were also as sensitive and specific at discriminating people with SZ from HC as the TMT. These results are in line with previous research showing poorer cognitive performance on the TMT in individuals with schizophrenia compared to controls^28,31^.

The present study is one of the first of its kind investigating the acceptability, validity and reliability of using a smartphone app to measure cognitive performance in schizophrenia^22^. Notably, this study provides initial validity for cognitive assessments on a smartphone app aiming to measure processing speed and executive functions in schizophrenia. While other studies have reported cognitive testing via surveys or tests on smartphones^32–34^, there is a need for more research using smartphone app EMAs of various cognitive domains in schizophrenia^35^. Our results show that the JTT hold similar psychometric properties compared to the TMT.

The ICC analyses indicated moderate reliability of the JTT over time which is similar to other computerized cognitive batteries^36,37^, but below what is observed with some other pen-and-paper standardized cognitive tests^5^. Moreover, reliability coefficients are often modest or not reported for EMAs and smartphone-based cognitive tests^9,22,38,39^; however, it is a critical piece of information required in the validation of a new cognitive test. Moving forward, further steps should be taken to ensure participants are completing the JTT in a quiet environment without distractions. For future applications of the JTT, we would like to implement a pop-up check-in that asks participants if they felt distracted while completing the tests, if they felt well-rested, and if they felt actively engaged in the tests. We could therefore exclude any data points that do not meet these criteria which would likely improve the reliability of our measures over time. We believe that although we observed modest reliability over time, the current findings are the first and critical step to establishing valid and reliable smartphone-based cognitive assessments that can be used in future research, as well as in clinical settings to improve current assessments of cognition^35^.

When exploring the impact of positive, negative, and general symptoms of schizophrenia on JTT performance, no relationships survived multiple comparisons. A few studies have investigated the associations between processing speed and executive functions, and clinical symptoms in schizophrenia. For instance, Laere and colleagues found that both clinical status and duration of illness were not associated with TMT performance^28^. Berman and colleagues observed a relationship between negative symptoms and the TMT A but not B, and did not find any associations between the TMT and positive symptoms^40^. In another study, only a relationship between the PANSS negative subscore and executive functions was observed, and there was no relationship between the general symptoms subscore and any cognitive domains measured, including executive functions^41^. More research is needed to clarify the associations between these cognitive domains and clinical symptoms of schizophrenia. Further longitudinal investigations using the JTT and weekly symptom assessments could be of great interest.

Significant practice effects were not observed on either Part A or B of the JTT over the three-month longitudinal portion of the study. The TMT has been reported to show practice effects^42^ which has previously tempted other researchers to develop alternate test forms. As practice effects can be the result of increased task familiarity through procedural learning^43^, the fact that we can create endless variations of the organization of the jewels on the JTT is a clear advantage of the smartphone version over any pen-and-paper version of the TMT^5^. The absence of significant practice effects in our results suggests that the JTT could be used remotely for a period of many weeks. However, it is possible that no significant practice effects were observed due to the specific number of completions observed over the 3-month longitudinal study period. Yet, most participants still completed the JTT remotely several times. It is also important to note that our study did not compensate participants for each weekly EMA completion, which likely explains the lower number of remote longitudinal completions observed here in comparison to the previous study using an EMA to measure cognition in schizophrenia^22^. We chose not to compensate participants for each weekly EMA completion as we did not want to introduce confounding variable of extrinsic motivation, which would not be replicated in day-to-day personal use or in clinical settings.

In addition, our results indicate that changes in self-reported mood over time could influence processing speed as measured by the smartphone JTT A. Previous research has shown that greater depressive symptoms are related to impaired processing speed and executive functions^44^, and EMAs like the JTT could make tracking these effects in real-time possible. Previous research has also revealed that processing speed measured by the TMT A has been shown to be affected by disturbed sleep in people with schizophrenia^45^ and healthy individuals^46,47^. Further research is needed to clarify the associations between self-reported mood and subjective sleep quality, and processing speed and executive function. While exploratory, our results provide some evidence that processing speed measured over time by the JTT may be susceptible to dynamic factors, such as lower self-report ratings of mood.

A major benefit of utilizing subjective surveys through a smartphone app is that they can be paired with the JTT and be used as an educational tool to provide key information to patients and their support teams such as tracking cognitive performance and symptomatology over time. Our study is a first step and future applications of the JTT could implement varying levels of testing frequency based on the specific research or clinical practice needs. In all, our results suggest that cognition could be influenced by variables that vary over time such as mood. Given these points, the development of remote, longitudinal cognitive assessments could allow the exploration of the effect of such variables on cognition over time.

The rapid transition of mental health resources to digital platforms is fueled by research findings, such as those presented in the current study, which provide evidence to support the acceptability, validity, and reliability of using ecological momentary smartphone cognitive assessments. While computerized cognitive assessments and more recently portable tablets have been shown to be an effective way of assessing cognition in people with schizophrenia^9,48^, smartphone assessments are becoming the new go-to tool as they can be used virtually anywhere^16,22,49,50^. Smartphone-based assessments, like the JTT, are becoming increasingly valued for cognitive assessment in psychiatric populations, given that they can provide real-time monitoring of cognition from anywhere at any time. Considering the steady rise of smartphone ownership and daily-usage, smartphone apps may be the key resource for reaching populations with limited access to mental health services^16^.

With further validation and research, smartphone apps have the potential to provide more accessible, time-oriented, and ecological assessments of cognition from an individual’s day-to-day environment. Future research could explore the impact of positive and negative symptoms, physical activities, and social interactions on cognition over time using the JTT on the MindLAMP app. Moreover, further investigation is needed to assess the divergent validity of the JTT in schizophrenia. In essence, EMAs are able to capture fluctuations in cognition over time as assessments can be completed multiple times per day or week and over long periods of time. Hence, smartphone apps can show how external and dynamic factors such as clinical symptoms, exercise, sleep, stress, and environment may affect cognition on a daily basis and over time^15^.

In summary, our results provide initial evidence that the mindLAMP app is an accessible tool with moderate validity and test-retest reliability to assess cognition in people with schizophrenia. Our findings support the use of the JTT to identify poorer cognitive performance in individuals with schizophrenia. More work remains to be done in order to optimize the JTT data and determine its full potential as a clinical tool. Open-access smartphone apps, like mindLAMP, have the potential to enable accessible, digital assessments of cognition for individuals with schizophrenia in both research and clinical settings. Our results can be reproduced and the JTT are available at no cost^29^, thus we hope that others will seek to replicate, and expand upon our results. Advancements in smartphone EMAs of cognition, such as those in the present study, are not only promising for cognitive assessments in research but also critical in clinical settings to assist the schizophrenia population who deal with persistent and debilitating cognitive impairments.

Our results need to be interpreted with the consideration of some limitations. First, our sample size is relatively small which may affect the generalization of our observed effect and further studies with greater sample sizes are needed. Nonetheless, the current study provides initial evidence on the JTT acceptability, validity and reliability, as well as critical insight on the potential of using such smartphone-based assessments to measure cognition in schizophrenia. Our groups were also not perfectly matched, but we observed significant group differences even when correcting for age and sex. Since our sample ranged from ages 18–64, we observed a decline in cognitive performance on the JTT which could be related to regular ageing effects on cognitive abilities^27,51^, and differences in age and smartphone habits and usage^52^.

An additional limitation to note is that although we asked participants to complete the JTT in a quiet environment, this cannot be verified. Also, the ideal testing environment for one participant may be too noisy or too quiet for another participant. We encourage future research to implement a survey check-in after completing the JTT asking participants if they were distracted by anything, if they felt focused, if there was excessive noise, etc. Self-reported mood and subjective sleep quality surveys used were also not previously validated and these measures were exploratory. While previous research indicates that self-report measures of mood^53^ and subjective sleep quality^54^ collected through a smartphone can be valid, future research should implement established depression and sleep questionnaires such as the PHQ-9^55^ and the PSQI^56^. Additionally, we chose to counterbalance the Part A and B of the TMT and JTT at the in-lab visit to avoid any unknown practice effects of A over B and B over A. We acknowledge that this is not the standard in neuropsychological testing using the TMT. Finally, we also observed some significant differences in performance between smartphone and pen-and-paper modalities. Differences between these modalities such as tapping the numbers on a phone instead of drawing a line between them, and differences in the Part Bs of each test may explain the differences in performance observed. The differences observed between modalities could also suggest that the smartphone app and pen-and-paper versions may not be directly comparable.

## Methods

### Participants

We recruited a total of 76 participants, including 32 individuals with schizophrenia or schizoaffective disorder (SZ) and 44 healthy controls (HC). All SZ participants received a clinical diagnosis of schizophrenia or schizoaffective disorder from a psychiatrist and were recruited from the outpatient program at the Royal Ottawa Mental Health Centre (Ottawa, ON, Canada). The diagnosis and presence of psychotic symptoms was confirmed by checking electronic medical records and using the MINI International Neuropsychiatric Interview (M.I.N.I.). Controls were recruited from the community using print and social media advertisements.

Participants were included if they were between the ages of 18–65, inclusively, were able to speak and read English above the sixth-grade level and were able to provide informed consent. Individuals with SZ were required to be on stable medication for at least one month. Participants were excluded from the study if they had a neurological disorder or other medical condition affecting cognition, a history of substance abuse or dependence within the past 3 months or did not own a smartphone. Control participants were also excluded if they presented any past or present psychiatric diagnosis, or if they had a first degree relative with a diagnosis of schizophrenia, schizoaffective disorder, or major depression.

The protocol was approved by The Royal Ottawa Research Ethics Board (REB# 2018040) and all participants signed an informed consent form before beginning any study procedures. Participants were invited to a single in-lab assessment lasting ~1 h where they were asked to download the mindLAMP app to their smartphone. They were then asked to keep the app on their phone for an additional 3 months while filling out surveys and completing the JTT on a weekly basis. Participants were compensated monetarily for their time in the in-lab visit only. Ten HC were excluded from the analyses: seven for not meeting the inclusion/exclusion criteria as discovered during the clinical interview, two for technical problems with their smartphone, and one for not properly following instructions during the assessment. Two individuals with SZ were also excluded: one for a technical problem related to their smartphone, and one for not meeting the inclusion/exclusion criteria as discovered during the clinical interview.

Interquartile range (IQR)^57^ was then used to remove outliers from all baseline and longitudinal analyses as this method is less affected by extreme outlier values and is commonly used in other research using an EMA^58–60^. Any data points 1.5* IQR above the third quartile or below the first quartile on the JTT or the TMT were removed from the analyses. After excluding outliers, the final sample consisted of a total of 34 HC and 29 SZ participants. From this sample, a total of 34 HC and 26 SZ participants had good quality data for both JTT and TMT and were kept for the in-lab baseline analysis. A total of 22 SZ and 23 HC had at least one good quality remote observation for the JTT A, and 20 SZ and 22 HC had at least one for the JTT B.

### In-lab clinical assessment

The M.I.N.I., based on the DSM-V^61^, was used to confirm the diagnosis of people with SZ, and to ensure that HC participants did not have an undisclosed past or present mental illness. The Positive and Negative Syndrome Scale (PANSS)^62^ was used to measure the severity of positive, negative, and general symptoms experienced in the last week in SZ participants. The chlorpromazine (CPZ) equivalent daily dose was calculated for all antipsychotic medications taken by each SZ participant^63^.

### In-lab cognitive assessment

All in-lab assessments were performed in a quiet testing room. Instructions and short practice trials were given before each assessment. Pen-and-paper and smartphone cognitive assessments were counterbalanced to avoid order effects. Additionally, the order of Part A and B for each test was counterbalanced.

The TMT^25,26^ Part A is a measure of processing speed where participants must connect the numbers 1 to 25 in chronological order by drawing a continuous line. Similarly, the TMT Part B measures executive functioning where participants must alternate between connecting a series of numbers (1–12) and letters (A-L) in sequential order (i.e., 1-A-2-B-3-C…). Participants were instructed to complete each test as quickly as possible without making any mistakes and were advised that if they made a mistake they would be stopped and instructed to start from the preceding correct number. Time to complete the test, in seconds, was used as a measure of cognitive performance for both Parts A and B.

Similar to the TMT, the JTT involve a Part A and B measuring processing speed and executive functioning, respectively. In Part A (Fig. 1a), participants were asked to tap the jewel-shaped numbers on their screen in sequential order from 1 to 25 as fast as possible without making any mistakes. Part B (Fig. 1b) involves alternating between two different shapes of the same number while tapping the numbers in sequential order (i.e., 1 shape-A, 1 shape-B, 2 shape-A, 2 shape-B…). Mistakes were tracked by the app with an addition of two seconds to the participant’s time, resulting in a poorer score. Completion time for the JTT was automatically obtained by the app and was used as a measure of cognitive performance.

### Remote longitudinal assessments

**Fig. 5:**
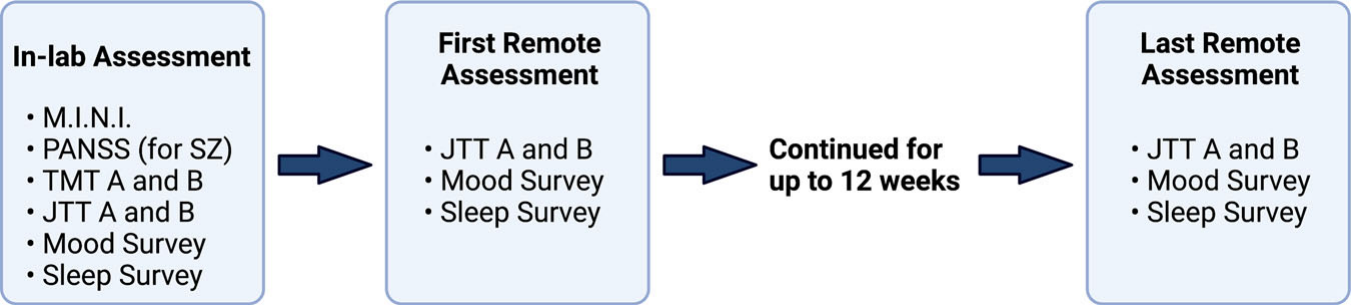
Timeline of the study procedures. M.I.N.I MINI International Neuropsychiatric Interview, PANSS Positive and Negative Syndrome Scale, TMT Trail Making Tests, JTT Jewels Trail Tests. The time of the last remote assessment differs per participant based on the number of times they completed the JTT A and B over the 12-week study period.

Participants were asked to keep the mindLAMP app on their phones for three months and to complete the JTT A and B as well as to fill out self-report mood and subjective sleep quality surveys weekly post-in-lab assessment (see Fig. 5). Participants received a notification once per week during the 3-month longitudinal study period to remind them to complete the EMA (i.e., 12 times), however, were told they could complete the EMA as many times as they wanted. They were also instructed to complete the JTT alone in a quiet space where they could focus on the task. Participants were asked to rate their mood and subjective sleep quality (i.e., “On a scale from 1–10 (1 = low; 10 = high) how was your mood today?”, “On a scale from 1–10 (1 = low; 10 = excellent) how was your sleep last night?”).

### Statistical procedures

All data were analyzed using R (version 1.2.5033). All analyses performed were two-sided, with a *p*-value threshold of .05 to determine significance, and Bonferroni’s corrections were applied for correcting for multiple comparisons.

Descriptive statistics for demographic and clinical variables were performed. Independent samples *t*-tests were used to determine whether there was a significant difference in age between groups. Chi-squares were also performed to determine whether there were significant differences in sex and smartphone usage between groups.

To determine how accessible the mindLAMP app was for our participants, we reported on the number of people who reached out to us with an initial interest for the study but did not participate and the associated reasons. We also reported the range and average number of times the participants used the app at home in the three-month longitudinal period.

Pearson’s correlations were used to assess the convergent validity between participant in-lab cognitive performance on the JTT and on the TMT, as well as for the last remote JTT and the in-lab JTT and TMT assessments. Post-hoc general linear models were then performed to investigate potential between-group interactions (SZ vs. HC) on these associations, as well as differences in completion time between modalities (smartphone vs. pen-and-paper).

Intraclass correlations (ICC) were used to examine the variability in cognitive scores within participants over time by comparing the following time points for each participant: the in-lab assessment completion time, as well as the first and last remote completion times. Only participants who successfully completed the in-lab assessment as well as a minimum of two remote assessments (i.e., first and last remote completion times) were included in these analyses (16 SZ and 21 HC for the JTT A; 17 SZ and 21 HC for the JTT B). The reliability analysis used an absolute agreement, two-way mixed-effects model to calculate the ICC for the JTT A and B independently^64^. ICC interpretation was determined based on Koo and Li (2016) where moderate reliability is defined as ICC values between 0.50 and 0.75^64^. Percent of overall group variance between the in-lab assessment and first-remote assessment completion times for the JTT A and B were also calculated.

A series of linear mixed models were used to determine whether there were significant differences between groups across all four in-lab cognitive tests, including age and sex as covariates. Effect sizes of the group comparisons are presented as standardized beta (*β*). Receiver operating characteristic curves analysis were also performed to compare sensitivity and specificity between both modalities in-lab (smartphone vs. pen-and-paper) in distinguishing people with schizophrenia from controls.

Linear mixed models were used to investigate practice effects on the JTT over time. The number of completion times for each test was the independent variable, cognitive performance was the dependent variable, and subject ID (intercept) was entered as a random factor. Similar linear mixed models were also used to examine how self-reported mood and subjective sleep quality may influence cognitive performance over time, with mood and sleep survey ratings as the independent variables for each model. Post-hoc linear mixed models were then performed to investigate potential between-group interactions (SZ vs. HC) on these associations, with age and sex as covariates.

Finally, Pearson’s correlations were used to explore the potential effect of screen size (measured in inches; obtained from manufacturer’s website), as well as symptom severity (measured by the positive, negative, and general subscores of the PANSS) on the in-lab JTT performance. The same methods were used to explore the relationship between the number of remote completions of the JTT and demographic variables including, age and years of education. Independent t-tests were also used to explore the potential effects of sex and group (SZ and HC) on the number of remote completions of the JTT.

### Reporting summary

Further information on research design is available in the Nature Research Reporting Summary linked to this article.

## Supplementary information

Supplementary Information

Reporting Summary

## Data Availability

The datasets generated during and/or analysed during the current study are available from the corresponding author on reasonable request.

## Code availability

The computer codes used to generate the results presented in this paper are available from the corresponding author on reasonable request.
