# Supplementary material for: Validation of an ecological momentary assessment to measure processing speed and executive function in schizophrenia
Source: NPJ Schizophr. 2021 Dec 21;7:64. doi: 10.1038/s41537-021-00194-9 (PMC8692600; doi:10.1038/s41537-021-00194-9)
Supplement: Supplementary file 1 — Supplementary Information [file 41537_2021_194_MOESM1_ESM.pdf]

## Supplementary Methods

Demographic and clinical data from the baseline in-lab sample are presented in Supplementary Table 1. We observed no statistically significant differences between our groups in terms of age, sex, and smartphone use.

**Supplementary Table 1.** *Demographic and Clinical Data in Schizophrenia and Control Groups from the baseline in-lab sample.*

| | Schizophrenia Group (*N* = 26) | Control Group (*N* = 34) | *p*-value |
|---|---|---|---|
| Age (years) | | | .10 |
| Mean (*SD*) | 37.2 (11.5) | 32.3 (11.3) | |
| Median [Min, Max] | 36.0 [20.0, 64.0] | 29.0 [18.0, 59.0] | |
| Sex | | | .13 |
| Male | 21.0 (80.8 %) | 20.0 (58.8 %) | |
| Female | 5.0 (19.2 %) | 14.0 (41.2 %) | |
| Smartphone Use | | | .85 |
| Every day of the week | 25.0 (96.4%) | 33.0 (97.1 %) | |
| 6 or less days per week | 1.0 (3.6%) | 1.0 (2.9 %) | |
| PANSS Positive Score | | | |
| Mean (*SD*) | 15.1 (5.4) | | |
| Median [Min, Max] | 15.0 [7.0, 28.0] | | |
| PANSS Negative Score | | | |
| Mean (*SD*) | 16.9 (6.9) | | |
| Median [Min, Max] | 15.0 [8.0, 33.0] | | |
| PANSS General Score | | | |
| Mean (*SD*) | 30.9 (7.9) | | |
| Median [Min, Max] | 30.0 [20.0, 52.0] | | |
| CPZ-equivalence | | | |
| Mean (*SD*) | 519.4 (419.3) | | |
| Median [Min, Max] | 392.0 [75.0, 2100.0] | | |

*Note. p*-value = probability value, *SD*= standard deviation, PANSS= Positive and Negative Syndrome Scale, CPZ-equivalence= chlorpromazine-equivalence. CPZ-equivalence was calculated for all antipsychotics based on the equivalent oral dose of chlorpromazine.
