# Supplementary material for: Validation of an ecological momentary assessment to measure processing speed and executive function in schizophrenia
Source: NPJ Schizophr. 2021 Dec 21;7:64. doi: 10.1038/s41537-021-00194-9 (PMC8692600; doi:10.1038/s41537-021-00194-9)
Supplement: Supplementary file 2 — Reporting Summary [file 41537_2021_194_MOESM2_ESM.pdf]

nature research

Corresponding author(s): Synthia Guimond

Last updated by author(s): May 18, 2021

Reporting Summary

Nature Research wishes to improve the reproducibility of the work that we publish. This form provides structure for consistency and transparency in reporting. For further information on Nature Research policies, see our Editorial Policies and the Editorial Policy Checklist.

Please do not complete any field with "not applicable" or n/a. Refer to the help text for what text to use if an item is not relevant to your study.
For final submission: please carefully check your responses for accuracy; you will not be able to make changes later.

Statistics

For all statistical analyses, confirm that the following items are present in the figure legend, table legend, main text, or Methods section.

n/a	Confirmed	
☐	☒	The exact sample size (n) for each experimental group/condition, given as a discrete number and unit of measurement
☐	☒	A statement on whether measurements were taken from distinct samples or whether the same sample was measured repeatedly
☐	☒	The statistical test(s) used AND whether they are one- or two-sided<br>*Only common tests should be described solely by name; describe more complex techniques in the Methods section.*
☐	☒	A description of all covariates tested
☐	☒	A description of any assumptions or corrections, such as tests of normality and adjustment for multiple comparisons
☐	☒	A full description of the statistical parameters including central tendency (e.g. means) or other basic estimates (e.g. regression coefficient) AND variation (e.g. standard deviation) or associated estimates of uncertainty (e.g. confidence intervals)
☐	☒	For null hypothesis testing, the test statistic (e.g. F, t, r) with confidence intervals, effect sizes, degrees of freedom and P value noted<br>*Give P values as exact values whenever suitable.*
☒	☐	For Bayesian analysis, information on the choice of priors and Markov chain Monte Carlo settings
☒	☐	For hierarchical and complex designs, identification of the appropriate level for tests and full reporting of outcomes
☐	☒	Estimates of effect sizes (e.g. Cohen's d, Pearson's r), indicating how they were calculated

Our web collection on statistics for biologists contains articles on many of the points above.

Software and code

Policy information about availability of computer code

Data collection	Smartphone data was collected from participants' smartphones using the mindLAMP app.
Data analysis	All analyses reported in this study used the statistical software R (version 1.2.5033).

For manuscripts utilizing custom algorithms or software that are central to the research but not yet described in published literature, software must be made available to editors and reviewers. We strongly encourage code deposition in a community repository (e.g. GitHub). See the Nature Research guidelines for submitting code & software for further information.

Data

Policy information about availability of data

All manuscripts must include a data availability statement. This statement should provide the following information, where applicable:
- Accession codes, unique identifiers, or web links for publicly available datasets
- A list of figures that have associated raw data
- A description of any restrictions on data availability

The datasets generated during and/or analysed during the current study are available from the corresponding author on reasonable request.

Field-specific reporting

Please select the one below that is the best fit for your research. If you are not sure, read the appropriate sections before making your selection.

☐ Life sciences ☒ Behavioural & social sciences ☐ Ecological, evolutionary & environmental sciences

Behavioural & social sciences study design

All studies must disclose on these points even when the disclosure is negative.

Study description	Quantitative case control study.
Research sample	Individuals with a diagnosis of schizophrenia or schizoaffective disorder from the schizophrenia outpatient program at The Royal Ottawa Mental Health Centre (Ottawa, Canada). Healthy controls were recruited from the community in the Ottawa, ON area. Both samples are of males and females ages 18-65. The diagnosis sample was chosen as there is minimal research on using smartphone cognitive testing apps to assess specific cognitive domains in people with schizophrenia.
Sampling strategy	Both stratified and convenience sampling were used. No sample size calculations were performed given the pilot nature of this study.
Data collection	All data were collected by a trained research assistant and were recorded both with pen-and-paper and computer using the Royal Ottawa Mental Health Centre's secure RedCap data collection server.
Timing	Data collection began in January 2019 and ended in June 2020.
Data exclusions	Ten controls were excluded from the analyses: seven for not meeting the inclusion/exclusion criteria as discovered during the clinical interview, two for technical problems with their smartphone, and one for not properly following instructions during the assessment. Two individuals with SZ were excluded from the analyses: one for a technical problem related to their smartphone, and one for not meeting the inclusion/exclusion criteria as discovered during the clinical interview. An additional four participants with SZ were excluded from the analyses for being outliers (with cognitive performance scores > 3 standard deviations from the mean). From the longitudinal sample, any data points from the JTT A or B that were 1.5* IQR above the third quartile or 1.5* IQR below the first quartile in each group were removed from the analyses. A total of 12 JTT A and 15 JTT B data points were excluded from the longitudinal analyses. The ICC analyses were performed in a subsample of 39 participants who completed at minimum two remote assessments of the JTT A and B.
Non-participation	Six individuals chose not to participate in the study as they did not want to download the smartphone app to their phone. All participants who signed a consent form completed the in-lab assessment, however; 15 participants did not complete any JTT A remote assessments and 17 participants did not complete any JTT B remote assessments.
Randomization	N/A

Reporting for specific materials, systems and methods

We require information from authors about some types of materials, experimental systems and methods used in many studies. Here, indicate whether each material, system or method listed is relevant to your study. If you are not sure if a list item applies to your research, read the appropriate section before selecting a response.

Materials & experimental systems

n/a	Involved in the study	
☒	☐	Antibodies
☒	☐	Eukaryotic cell lines
☒	☐	Palaeontology and archaeology
☒	☐	Animals and other organisms
☐	☒	Human research participants
☒	☐	Clinical data
☒	☐	Dual use research of concern

Methods

n/a	Involved in the study	
☒	☐	ChIP-seq
☒	☐	Flow cytometry
☒	☐	MRI-based neuroimaging

Human research participants

Policy information about studies involving human research participants

Population characteristics	See above.

Recruitment	Healthy controls were recruited from the community via print and social media advertisements. Individuals with schizophrenia were recruited from the outpatient program at the Royal Ottawa Mental Health Centre in Ottawa, ON, Canada.
Ethics oversight	The study protocol was approved by the Royal Ottawa Mental Health Centre Research Ethics Board (REB# 2018040).

Note that full information on the approval of the study protocol must also be provided in the manuscript.

This checklist template is licensed under a Creative Commons Attribution 4.0 International License, which permits use, sharing, adaptation, distribution and reproduction in any medium or format, as long as you give appropriate credit to the original author(s) and the source, provide a link to the Creative Commons license, and indicate if changes were made. The images or other third party material in this article are included in the article's Creative Commons license, unless indicated otherwise in a credit line to the material. If material is not included in the article's Creative Commons license and your intended use is not permitted by statutory regulation or exceeds the permitted use, you will need to obtain permission directly from the copyright holder. To view a copy of this license, visit http://creativecommons.org/licenses/by/4.0/
